# Validation of the ligase detection reaction fluorescent microsphere assay for the detection of *Plasmodium falciparum* resistance mediating polymorphisms in Uganda

**DOI:** 10.1186/1475-2875-13-95

**Published:** 2014-03-14

**Authors:** Sheila Nankoberanyi, George W Mbogo, Norbert P LeClair, Melissa D Conrad, Patrick Tumwebaze, Stephen Tukwasibwe, Moses R Kamya, Jordan Tappero, Samuel L Nsobya, Philip J Rosenthal

**Affiliations:** 1 Infectious Diseases Research Collaboration, Kampala, Uganda; 2 Department of Medicine, University of California, Box 0811, San Francisco, CA 94143, USA; 3 Makerere University, Kampala, Uganda; 4 Centers for Disease Control and Prevention, Center for Global Health, Atlanta, GA, USA

**Keywords:** *Plasmodium falciparum*, Malaria, Fluorescent microsphere assay, Drug resistance, Polymorphisms

## Abstract

**Background:**

Malaria remains a major public health problem, and its control has been hampered by drug resistance. For a number of drugs, *Plasmodium falciparum* single nucleotide polymorphisms (SNPs) are associated with altered drug sensitivity and can be used as markers of drug resistance. Several techniques have been studied to assess resistance markers. The most widely used methodology is restriction fragment length polymorphism (RFLP) analysis. The ligase detection reaction fluorescent microsphere (LDR-FM) assay was recently shown to provide high throughput assessment of *P. falciparum* SNPs associated with drug resistance. The aim of this study was to validate the reliability and accuracy of the LDR-FM assay in a field setting.

**Methods:**

For 223 samples from a clinical trial in Tororo, Uganda in which *P. falciparum* was identified by blood smear, DNA was extracted from dried blood spots, genes of interest were amplified by PCR, amplicons were analysed by both RFLP and LDR-FM assays, and results were compared.

**Results:**

SNP prevalence (wild type/mixed/mutant) with RFLP analysis was 8/5/87% for *pfcrt* K76T, 34/37/29% for *pfmdr1* N86Y, 64/17/19% for *pfmdr1* Y184F, and 42/21/37% for *pfmdr1* D1246Y. These prevalences with the LDR-FM assay were 7/5/88%, 31/24/45%, 62/20/18%, and 48/19/33% for the four SNPs, respectively. Combining mixed and mutant outcomes for analysis, agreement between the assays was 97% (K = 0.77) for *pfcrt* K76T, 79% (K = 0.55) for *pfmdr1* N86Y, 83% (K = 0.65) for *pfmdr1* Y184F, and 91% (K = 0.82) for *pfmdr1* D1246Y, with most disagreements due to discrepant readings of mixed genotypes.

**Conclusion:**

The LDR-FM assay provides a high throughput, relatively inexpensive and accurate assay for the surveillance of *P. falciparum* SNPs associated with drug resistance in resource-limited countries.

## Background

Malaria, especially that caused by *Plasmodium falciparum*, remains one of the most important infectious diseases in the world. The treatment and control of malaria has been greatly hampered by parasite resistance to available drugs. With widespread resistance to older drugs, artemisinin-based combination therapy (ACT) is now the standard of care for the treatment of falciparum malaria. In Uganda, artemether-lumefantrine (AL) was introduced as first-line treatment for uncomplicated malaria in 2006 [1].

Mechanisms of resistance to anti-malarial drugs are incompletely understood. For chloroquine and amodiaquine, the K76T mutation in the *pfcrt* gene, which encodes a putative drug transporter, is the principal mediator of resistance [2]. Polymorphisms in another gene, *pfmdr1*, which encodes a protein homologous to transporters that mediate drug resistance in other organisms, modulate levels of sensitivity to multiple drugs [3]. In Africa, the *pfmdr1* N86Y, Y184F and D1246Y polymorphisms are common, and the 86Y and 1246Y mutations are associated with decreased sensitivity to chloroquine and amodiaquine [4-6]. Interestingly, wild type sequences at these same alleles lead to decreased sensitivity to artemisinins and the ACT partner drugs lumefantrine and mefloquine [5,7,8]. Given the limited arsenal of effective ACT and early signs of artemisinin resistance in Southeast Asia [9,10], there is a need for efficient surveillance of Ugandan parasites for genetic polymorphisms that may mediate resistance to the most important anti-malarial drugs.

Several techniques have been developed to detect single nucleotide polymorphisms (SNPs) associated with *P. falciparum* drug resistance. The most widely used is restriction fragment length polymorphism (RFLP) analysis [11], which is reliable, but fairly expensive and labour intensive. Other relatively low throughput methodologies include direct DNA sequencing, mutation-specific PCR [12], dot-blot probe hybridization [13], molecular beacons [14], and single-nucleotide primer extension [15]. Other techniques that have provided improved throughput include polymorphism-specific microarrays [16], melting curve analysis [17,18] and quantitative PCR [19-21]. Each of these methodologies has challenges, especially in resource-limited settings, including cost and availability of required instruments.

The ligase detection reaction fluorescent microsphere (LDR-FM) assay allows multiplex assessment of multiple *P. falciparum* SNPs [22]. It was recently shown that this assay is accurate and also less expensive and less labour intensive compared to RFLP analysis [23]. This new study compared the LDR-FM assay with RFLP analysis for the detection of key resistance-mediating *P. falciparum* SNPs in samples from a Ugandan clinical trial, with assays performed in parallel at a Ugandan laboratory.

## Methods

### Samples for analysis

Control parasite DNA was obtained from the Malaria Research and Reference Reagent Resource Center. Field samples were from a longitudinal anti-malarial drug efficacy trial in Tororo, Uganda, the details of which have been published [24]. Briefly, 351 children aged four to 12 months were enrolled and randomized to receive either AL or dihydroartemisinin-piperaquine (DP) for each episode of uncomplicated malaria between 2007 and 2012.

All first episodes of falciparum malaria and all recurrent malaria episodes presenting 84 or more days after prior treatment were studied. Parasite densities were estimated by counting the number of asexual parasites per 200 white blood cells and calculating parasites per μL, assuming a white blood cell count of 8,000 cells/μL. The study was approved by the Uganda National Council of Science and Technology and the Institutional Review Boards of Makerere University College of Health Sciences and the University of California, San Francisco, USA.

### RFLP analysis

DNA was extracted from filter paper with Chelex resin [25] and alleles were identified by nested PCR (see Additional file 1 for primers) followed by RFLP analysis, as previously described [26,27]. Briefly, regions of interest were amplified, PCR products were treated with polymorphism-specific restriction endonucleases (*Apo*I for *pfcrt* K76T, *Afl*III for *pfmdr1* N86Y, *Dra*I for *pfmdr1* Y184F, and *Bgl*II for *pfmdr1* D1246Y), and the sizes of products were characterized by agarose gel electrophoresis to distinguish wild type, mutant and mixed alleles based on comparison with control reference strain DNA.

### LDR-FM analysis

The extracted DNA was amplified by PCR (see Additional file 2 for primers), as previously described [23]. The amplicons were subjected to multiplex ligase detection reactions (see Additional file 3 for primers) in which bead-specific oligonucleotides and biotin were added, ligation products were hybridized to magnetic beads, and polymorphism prevalences were assessed fluorometrically in a multiplex format using a Magpix instrument (Luminex). Genotypes were determined based on comparisons with controls, with a minimum threshold of 400 MFI for each reaction and correction factors for each SNP, as previously described [23].

### Statistical analysis

Correlation of results between assays was assessed using kappa statistics.

## Results

### Analysis of *plasmodium falciparum* reference strains

The goal of this study was to validate the LDR-FM assay, using samples from a recent clinical trial, with assays performed at a molecular laboratory in Kampala, Uganda. The LDR-FM assay was first evaluated with DNA from laboratory adapted *P. falciparum* strains 3D7, 7G8, DD2 and V1/S. Three SNPs in *pfmdr1* that are common in Uganda and a five-amino acid haplotype in *pfcrt* that distinguishes chloroquine-sensitive and -resistant parasites with different geographic backgrounds were assessed [28]. Sequence determinations at all studied polymorphisms were straightforward, with background readings for unidentified SNPs five to ten-fold lower than the readings for correct identifications (Table 1).

**Table 1:** **LDR-FM readings for four SNPs in *Plasmodium falciparum *** **reference strains**

**Strain**	***pfmdr 1***						***pfcrt***		
	**86 N**	**86Y**	**184Y**	**184 F**	**1246D**	**1246Y**	**CVMNK**	**CVIET**	**SVMNT**
3D7	**4,325**	119	**3,316**	251	**3,720**	191	**1,330**	237	281
7G8	**3,680**	187	197	**4,087**	123	**1,452**	458	741	**3,133**
DD2	214	**1,689**	**3,810**	256	**4,773**	153	210	**2,660**	214
V1/S	126	**4,018**	**3,679**	284	**3,599**	193	309	**2,647**	315

Values shown are uncorrected mean fluorescence readings for three to five assays, each run in triplicate. For *pfcrt*, the haplotype represents amino acids 72–76. Readings representing the known sequences at each allele are in bold type.

### Analysis of clinical samples

**Figure 1:**
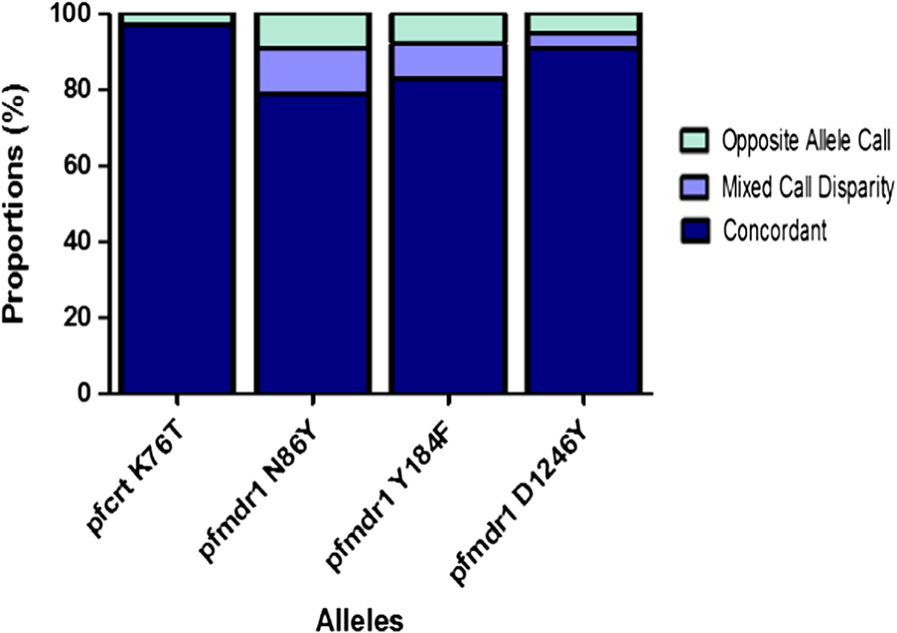
**Agreement between RFLP and LDR-FM assays.** Results are shown for samples with readings from both assays at the indicated alleles.

A total of 230 smear-positive clinical samples were analysed by both RFLP and LDR-FM assays. The characteristics describing these samples are shown in Table 2. Representative uncorrected fluorescence data for ten samples showed generally good discrimination of wild type, mixed and mutant genotypes (Table 3). Results were available from 223 samples for both assays and these were used for comparisons. Assays were unsuccessful for six RFLP and zero LDR-FM assays for *pfcrt*, seven RFLP and seven LDR-FM assays at *pfmdr1* 86, seven RFLP and seven LDR-FM assays at *pfmdr1* 184, and three RFLP and one LDR-FM assays at *pfmdr1* 1246. SNP prevalences measured by RFLP analysis and with the LDR-FM assay were similar at all studied alleles (Table 4). Agreement between the assays was good, although results varied between the studied alleles (Figure 1). Most discrepancies were due to a mixed reading with one assay, compared to a pure mutant or wild type reading with the other assay. Combining mixed and mutant outcomes for analysis, agreement between the assays was 97% (K = 0.77) for *pfcrt* K76T, 79% (K = 0.55) for *pfmdr1* N86Y, 83% (K = 0.65) for *pfmdr1* Y184F, and 91% (K = 0.82) for *pfmdr1* D1246Y.

**Table 2:** Baseline characteristics of the samples utilized in this study

**Characteristic**	**Treatment arm**	
	**AL (n = 101)**	**DP (n = 129)**
Mean age, months ± SD	30.4 ± 14	30.4 ± 14
Mean parasite density, geometric mean cells/μl (IQR)	18,534 (8160–74,277)	14,164 (4720–60,800)
Mean time since last episode of malaria, days	174	166
Year of collection	2008-2012	2008-2012
Mean time between collection and assay (range)	3.7 years (1–5)	3.6 years (1–5)

*AL* artemether-lumefantrine; *DP* dihydroartemisinin-piperaquine; *IQR* inter-quartile range; *SD* standard deviation.

**Table 3:** Representative LDR-FM results for ten clinical samples

**Sample**	**Parasite density (per μl)**	***pfmdr1***						***pfcrt***		
		**86 N**	**86Y**	**184Y**	**184 F**	**1246D**	**1246Y**	**CVMNK**	**CVIET**	**SVMNT**
1	12,560	**3,386**	106	**3,600**	209	**2,018**	101	**1,235**	231	174
2	74,080	**1,849**	**1,885**	**2,083**	**2,284**	**3,286**	309	811	**1,678**	195
3	35,120	**812**	**2,937**	177	**4,031**	**4,140**	365	232	**2,115**	195
4	8,960	**3,761**	188	**2,879**	**3,361**	**4,536**	164	207	**2,311**	214
5	24,560	**4,081**	125	**4,068**	322	**3,386**	93	162	**2,438**	163
6	1,440	143	**3,777**	**4,024**	292	115	**2,065**	222	**2,430**	164
7	25,520	**1,502**	**3,754**	**3,928**	**1,788**	**2,496**	**1,881**	304	**2,440**	163
8	36,064	**2,829**	**2,846**	311	**5,227**	**3,148**	937	**1,119**	**1,610**	211
9	18,320	**2,677**	132	**3,154**	555	**4,254**	143	**1,097**	**1,262**	243
10	14,840	**4,121**	100	**3,686**	267	196	**1,715**	**1,385**	317	231

Values shown are uncorrected mean fluorescence readings. Readings representing the sequence call at each allele after correction by subtraction of background are in bold type.

**Table 4:** Genotype results as analysed by RFLP and LDR-FM assays

**Genotype**	***pfcrt K76T***		***pfmdr1 N86Y***		***pfmdr1 Y184F***		***pfmdr1 D1246Y***	
	**RFLP**	**LDR-FM**	**RFLP**	**LDR-FM**	**RFLP**	**LDR-FM**	**RFLP**	**LDR-FM**
**Wild type**	17 (8%)	16 (7%)	76 (34%)	69 (31%)	143 (64%)	139 (62%)	94 (42%)	107 (48%)
**Mixed**	12 (5%)	11 (5%)	83 (37%)	53 (24%)	38 (17%)	45 (20%)	47 (21%)	42 (19%)
**Mutant**	194 (87%)	196 (88%)	64 (29%)	101 (45%)	42 (19%)	39 (18%)	82 (37%)	74 (33%)

The numbers of samples with each genotype and percentages of total assignments are shown.

## Discussion

The aim of this study was to validate the LDR-FM assay for the detection of SNPs associated with anti-malarial drug resistance in a Ugandan laboratory. A total of 230 smear-positive clinical samples were analysed for four SNPs associated with anti-malarial drug resistance using RFLP and LDR-FM assays, and results were compared. It is important to note that both systems have potential for errors in misclassification, in particular due to challenges in distinguishing pure and mixed genotypes at each allele of interest. As seen in a prior study, SNP prevalences measured by RFLP and LDR-FM analyses were similar at the studied alleles, with most discrepancies between assays due to different calls for mixed genotypes, and with DNA sequencing showing that for 8 of 9 discrepant readings the LDR-FM result was correct [23].

Several techniques have been assessed to detect SNPs associated with *P. falciparum* anti-malarial drug resistance, each with potential advantages and disadvantages in a resource-limited setting. Of particular importance is availability of equipment necessary for high throughput methods. The LDR-FM assay requires an expensive device, but this unit can provide a range of assays, including measurements of serum cytokine levels and antibody reactivity, suggesting that it will be of value for many field-based laboratories. Reagent costs for the LDR-FM assay are also significant, but this is the case with all molecular assays, and recent estimates suggested that the assay provides results at about half the reagent costs of RFLP assessments [23]. Further, throughput was estimated to be five to ten-fold better with the LDR-FM assay compared to RFLP analysis, offering large potential savings in personnel costs.

## Conclusions

The LDR-FM assay, when performed in a developing-world laboratory, provided accurate assessment of SNPs associated with anti-malarial drug resistance. This method offers relatively high throughput and low cost, hence it will be used for large-scale surveillance of *P. falciparum* polymorphisms of interest in ongoing studies in Uganda [29]. For other field-based laboratories with access to Magpix instrumentation, this system may offer the most practical means of large-scale surveillance of *P. falciparum* genetic polymorphisms.

## Abbreviations

RFLP: Restriction fragment length polymorphism; LDR-FM: Ligase detection reaction fluorescent microsphere; SNP: Single nucleotide polymorphism; AL: Artemether-lumefantrine; DP: Dihydroartemisinin-piperaquine; ACT: Artemisinin-based combination therapy.

## Competing interests

All authors declare that there are no competing interests.

## Authors’ contributions

SN ran the LDR-FM assays, analysed the data and wrote the first draft of the manuscript. GWM ran the RFLP assays and helped with data analysis. NPL, MDC, PT, ST, and SLN provided advice and technical support for optimization of the LDR-FMA protocol in Uganda. MRK and JT oversaw design and completion of the clinical trial that provided samples for this study. PJR assisted in the design the study, obtained funding, and wrote the first draft of the manuscript. All authors contributed to preparation of this manuscript and read and approved the final draft.

## Supplementary Material

Additional file 1 PCR primers for RFLP analyses. Click here for file

Additional file 2 PCR primers for LDR-FM analyses. Click here for file

Additional file 3 LDR primers for LDR-FM analyses. Click here for file
