# Supplementary material for: Validation of the ligase detection reaction fluorescent microsphere assay for the detection of Plasmodium falciparum resistance mediating polymorphisms in Uganda
Source: Malar J. 2014 Mar 14;13:95. doi: 10.1186/1475-2875-13-95 (PMC4004386; doi:10.1186/1475-2875-13-95)
Supplement: Additional file 1 — PCR primers for RFLP analyses. [file 1475-2875-13-95-S1.docx]

### Additional file 1: PCR primers for RFLP analyses

| *Pfmdr-1* 86 & 184  1^st^ round | MDR-A1: 5’TGTTGAAAGATGGGTAAAGAGCAGAAAGAG3’  MDR-A3: 5’TACTTTCTTATTACATATGACACCACAAACA3’ |
| --- | --- |
| *Pfmdr-1* 86 & 184  2^nd^ round | MDR-A4: 5’AAAGATGGTAACCTCAGTATCAAAGAAGAG3’  MDR-A2: 5’GTCAAACGT GCA TTTTTTATTAATGACCAT TTA3’ |
| *Pfmdr-1* 1034, 1042 & 1246  1^st^ round | MDR-01: 5’AGAAGATTATTTCTGTAATTTGATACAAAAAGC3’  MDR-02: 5’ATGATTCGATAAATTCATCTATAGCAGCAA3’ |
| *Pfmdr-1* 1246  2^nd^ round | 1246F: 5’ATGATCACATTATATTAAAAAATGATATGACAAAT3’  MDR-02: 5’ATGATTCGATAAATTCATCTATAGCAGCAA3’ |
| Pfcrt 76  1^st^ round | 76-A: 5’GCG CGCGCATGGCTCACGTTTAGGTGGAG3'  76-B: 5'GGGCCCGGCGGATGTTACAAAACTATAGTTACC3' |
| Pfcrt 76  2^nd^ round | CQR-A: 5’TGTGCTCATGTGTTTAAACTT3’  CQR-B: 5’CAAAACTATAGTTACCAATTTTG3’ |
