# Supplementary material for: Validation of the ligase detection reaction fluorescent microsphere assay for the detection of Plasmodium falciparum resistance mediating polymorphisms in Uganda
Source: Malar J. 2014 Mar 14;13:95. doi: 10.1186/1475-2875-13-95 (PMC4004386; doi:10.1186/1475-2875-13-95)
Supplement: Additional file 2 — PCR primers for LDR-FM analyses. [file 1475-2875-13-95-S2.docx]

**Additional file** **2: PCR primers for LDR-FM analyses**

| **Target gene** | **Primer Name** | **Sequence** |
| --- | --- | --- |
| *Pfcrt 76* | CRQ-A | 5’TGT GCT CAT GTG TTT AAA CTT3’ |
|  | CRQ-B | 5’CAA AAC TAT AGT TAC CAA TTT TG3’ |
| *Pfmdr1 86 and*  *Pfmdr1 184* | F3 Forward | 5’TGT ATG TGC TGT ATT ATC AG3’ |
|  | F3 Reverse | 5’CTT ATT ACA TAT GAC ACC ACA3’ |
| *Pfmdr1 1246* | F5 Forward | 5’TAG AAG ATT ATT TCT GTA ATT T3’ |
|  | F5 Reverse | 5’CAA TGT TGC ATC TTC TCT TCC A3’ |
