# Supplementary material for: Validation of the ligase detection reaction fluorescent microsphere assay for the detection of Plasmodium falciparum resistance mediating polymorphisms in Uganda
Source: Malar J. 2014 Mar 14;13:95. doi: 10.1186/1475-2875-13-95 (PMC4004386; doi:10.1186/1475-2875-13-95)
Supplement: Additional file 3 — LDR primers for LDR-FM analyses. [file 1475-2875-13-95-S3.docx]

**Additional file** **3: LDR primers for LDR-FM analyses**

| **Primer** | **Bead** | **Sequence** |
| --- | --- | --- |
| 86 Common |  | /5Phos/CATGTTCTTTAATATTACACCAAA/3Bio/ |
| 86N | 12 | cataatcaatttcaactttctactAGGATTAATATCATCACCTAAATT |
| 86Y | 48 | aatcaacacacaataacattcataAGGATTAATATCATCACCTAAATA |
| 184 Common |  | /5Phos/TATTTGGTCATTAATAAAAAATGCA/3Bio/ |
| 184Y | 14 | aatttcttctctttctttcacaatGCCAGTTCCTTTTTAGGTTTATA |
| 184F | 63 | ctaaatcacatacttaacaacaaaGCCAGTTCCTTTTTAGGTTTATT |
| 1246 Common |  | /5Phos/ATCTTAGAAACTTATTTTCAATAG/3Bio/ |
| 1246D | 22 | caaacaaacattcaaatatcaatcAATATATGTGATTATAACTTAAGAG |
| 1246Y | 61 | aatctctacaatttctctctaataTAATATATGTGATTATAACTTAAGAT |
| Pfcrt 72-76 Common |  | /5Phos/AATTTTTGCTAAAAGAACTTTAAAC/3Bio/ |
| CVMNK | 19 | atactttacaaacaaataacacacATTTAAGTGTATGTGTAATGAATAA |
| CVIET | 15 | tacttctttactacaatttacaacATTTAAGTGTATGTGTAATTGAAAC |
| SVMNT | 77 | aataacaactcactatatcataacATTTAAGTGTAAGTGTAATGAATAC |

Lowercase nucleotides represent tag sequences added to the 5’ ends of each allele-speciﬁc LDR primer. Bead numbers represent unique codes for beads linked to each studied SNP. Definitions: Common**,** conserved sequence primer positioned immediately downstream from the allele-speciﬁc primer; /5Phos/, 5’ phosphorylation; /3Bio/, 3’ biotinylation**.**
